# J. B. Van Helmont's System of Medicine Compared with the More Remarkable Systems of Ancient and Modern Time; a Contribution to the History of the Development of Medical Theories; Together with a Sketch of a Theory of the Phenomena of Life

**Published:** 1843-10

**Authors:** 


### ART. XI.

*J. B. van Helmont's System der Medicin, verglichen mit den bedeutenderen Systemen älterer und neuerer Zeit; ein Beitrag zur Entwickelungs-geschichte medicinischen Theorien; nebst der Skizze einer Theorie der Lebenserscheinungen.* Von Dr. G. A. SPIESS.—*Frankfurt am Main*, 1840. 8vo, pp. 520.

*J. B. van Helmont's System of Medicine compared with the more remarkable Systems of ancient and modern Time; a contribution to the history of the development of medical theories; together with a sketch of a theory of the phenomena of life.* By Dr. G. A. SPIESS, Practising Physician.—*Frankfort on the Main*, 1840.

WE lately gave some account of the works of Paracelsus according to the estimate formed of them by Professor Marx, of Göttingen. By a happy coincidence an occasion is now afforded for a consideration of those of Van Helmont, who in the history of the reformation of medicine from the errors of the Greek and Arabian schools, stands next to Paracelsus in time, and equal or superior to him in dignity and learning. The title of this work so fully tells its scope, that we may proceed at once to the analysis of its contents, and, following the order of the learned author, we shall review, first, the general features of Van Helmont's life; secondly, his doctrines; thirdly, their relation to the views of his predecessors; and, fourthly, their relation to those of succeeding ages and of the present time.

I. JOHANNES BAPTISTA VAN HELMONT, Lord of Merode, Royenborch, Oerschot, and Pellines, was born in 1578, the scion of a noble Netherlands family. He was the youngest child of his father, who died in 1580; but he received, through the affection of his mother, a careful education, and in his seventeenth year had completed the course of philosophic instruction in the university of Louvain, and already called himself "helluo librorum." Many, perhaps, surpassed him in real knowledge, but few had equalled him in industry. He had studied earnestly mathematics, algebra, and astronomy; but when he learnt from Copernicus that the heavens turned in a manner altogether different from that which, in much labour, he had before been taught, astronomy fell at once into contempt with him, and he held it for an empty pretence of science. Such were already, too, his scepticism and diffidence, that he would not take his master's degree because he felt that he did not yet deserve even to be called a scholar; and he left the schools as places where the truth he earnestly longed after had never dwelt.

A rich canonry was offered him if he would devote himself to theology, but he dared not, as St. Bernard said, "live by the sins of men." The Jesuits for a time enslaved him by their philosophy, but at last, in all they taught, he could find but "empty straw." He studied Seneca and Epictetus; and, for a time, he suspected that the very sap of truth must be in ethics. He thought the Capucin monks were the true Christian stoics, and though, for his weak health's sake, he dared not enter their order, yet he was convinced that if he could but adopt, in their Christian completeness, all the principles of the stoical philosophy, he should reach both to the knowledge and the love of pure truth. The notion lasted till he had a strange dream. He seemed to be transformed into a huge empty bubble reaching from earth to heaven; over him hung a sword, below was an abyss of darkness; and he felt how the philosophy had made him like a great inflated being floating over the abyss of hell. Terrified at his having dared to attempt all things by his own will and power, he left the stoical philosophy as that which might do well enough for heathens, but to Christians must be hateful blasphemy. He felt that God alone could give wisdom, and that prayer was a better instrument for its attainment than self-willed study.

In his uncertainty what course of life he should pursue, Van Helmont turned now to the study of laws and government; but he soon found that what was called right depended only on the varying opinions of men, and he thought it would be mere loss of time to learn what this or that man had happened to command. At length, wearied with his vain search for truth and happiness, he took up Matthiolus and Dioscorides, thinking that nothing could be better for man than to admire God's goodness in the herbs, and to employ them all in their right uses. But it was presently clear to him that, since the time of Dioscorides, the study had not essentially advanced, and that, at the best, the therapeutic herbal was a very confused unsatisfying book.

This study, however, led Van Helmont into that of medicine, and he wondered whether there were a book in which he could learn its principles and rules; for he supposed that medicine, being a science and a good gift from heaven, would have its certain axioms. So he read the Institutes of Fuchsius and Fernelius, and he could not help laughing with himself at their poverty and weakness. Then he read through Galen twice and Hippocrates once; he read the whole of Avicenna and the other Greek and Arabian authors—in all, near six hundred books. With great industry, too, he set down all that seemed important; and, at the last, when he read through his notes, he found only reason to grieve that he had thrown away such mighty labour, for the poor ill-founded principles which he first read and laughed at contained nearly all that he had learnt.

He now determined to join a practising physician and see patients with him; but here, again, he found nothing but uncertainty; all the diseases which did not cease of themselves seemed to be classed among the incurable. Disgusted with medicine, and with himself for having, in spite of his noble origin and against the wish of his mother, consented to study it, Van Helmont gave his inheritances to a widowed sister, and his books to poor students, and determined to leave his country for ever, and to roam in foreign lands in the hope that God would yet guide his course for good.

But the more he fled from medicine, and the more obstinately he regarded it as mere trickery, the more occasion did he find for practising it, and the more marvellous were the cures he effected. In his travels, too, he met with a plain man who knew little more than the manipulations of pyrotechny, but who taught him so much chemistry that in two years he had made a whole store of mineral medicines, and could employ them all with marvellous success. This seems to have revived a hope that something might yet be done in medicine, and his essays encouraged him; for, on his way, many great persons asked his aid in their diseases, and they recovered in his hands; many times too he found pestilences raging in the towns he visited, and, moved with compassion for the poor wretches whose neighbours and physicians had alike deserted them, he frequented all their haunts, and found that while he was studying diseases his mere presence comforted and sometimes cured the sick. At length after ten years of study and of travel, in which he passed some time in England, he returned in 1609 to Vilvorden, near Brussels, where he determined to seclude himself for prayer and for the study of nature, and especially of pyrotechny. Here too he read the works of Paracelsus, which, though he at last discerned their many errors, made a deep impression on his mind. And hither, though he would have avoided it, numbers came from far and near for his advice.

The last thirty years of Van Helmont's life were thus spent in a retirement, in which his study and his meditations were interrupted only by his attendance upon those who needed his charity, or whom his fame had attracted to seek his aid. During this period too, he wrote nearly all his works, and they were published by his son after his death. He died in 1644, of some chest-affection. On his last day he wrote standing, for he could not lie down for fear of suffocation, to a friend in Paris, "praise and honour be to God for ever, whom it has pleased to take me from this world. As I think, my life will not continue more than four and twenty hours, for I have just suffered the first attack of fever from weakness and want of vital action, so I must conclude;" and his prophecy proved true.

This brief account of Van Helmont, in which his own language is for the most part imitated, may convey some notion of his character. Dr. Spiess, who must however be regarded rather as a panegyrist than as an impartial biographer, points out as its most striking features, acuteness of judgment enabling him to detect the most hidden weakness of the medical systems of his time, deep-felt sense of religion producing a genuine philanthropy, independence, and self-confidence. And these qualities Van Helmont unquestionably possessed; they are assigned to him even in the matter-of-fact estimates of Haller, and they will be more obvious in all that is said of his doctrines. But they were not unalloyed; vanity and personal ambition are as distinct in all his writings as these better qualities are. His sharp discernment of errors was rarely employed in testing the statements on which he supported his own system; in all that favoured his own views he was as credulous as any of his predecessors. Witness, for example, his belief in all the imaginary facts of natural magic, even to the tale of the transplanted nose falling off on the death of its original proprietor, his satisfaction with the results of his own medical practice, and his discontent with that of others. And again, if his religion produced philanthropy, so also it often degenerated into mere superstition; a belief in all the pretended miracles of the Romish church, a reliance upon dreams and the visions of a disordered fancy, an acquiescence in many of the absurdest mysticisms of the Neo-Platonists.

We proceed to an analysis of his systems of natural science and of medicine, for the two are so closely interwoven that they cannot separately be made intelligible. And here also we shall write in something like Van Helmont's style, displaying the system as in an uninterrupted discourse, and only asking the reader to bear in mind that the laws of gravitation, electricity, and chemical affinity, and nearly all the facts of organic physiology were unknown when the original was written.

II. Van Helmont's system of philosophy and medicine.

A. General considerations. The prime cause of all natural phenomena is not, as the Aristotelians and nearly all men maintain, "a principle of motion and rest in bodies, in which it exists *per se* and not *per accidens ;*" but nature is the command of God, by which each thing is what it is, and does that which it is commanded to do. Everything in nature has its origin from the direct union of matter, (the substratum of all natural things) and a *causa efficiens*, which is also named *causa seminalis, principium dirigens, faber, Vulcanus*, or in the widest sense, *Archæus ;* and into this Archæus, God has infused the knowledge of its own ends and habitudes. But the matter and the efficient cause must not be regarded as separate; they are in every natural being most intimately connected, and they determine its existence; for with their union a determinate *form*, or mode and capacity of existence,* is always given, which depends on the efficient cause.

Whenever, by the union of matter and an indwelling power, or internal efficient cause, a determinate form is given, there is also *life;* and thus all nature is enlivened, for in every natural being there is matter with a determinate plan of existence. Moreover, all life may be designated *light*, and in so far as it appears in determinate form, as formal or form-giving light (*lux formalis ;*) and further than this, we cannot apprehend the intimate essence of life, "for life in the abstract is the very God incomprehensible."

Diversities in the relative proportions of life or light, and of matter, in different beings do not alone constitute their differences. It is true that "certain forms shine, as in stones and minerals; and certain are resplendent with augmented light, as in plants; while others are even luminous, as in animals;" but "there are also as many *kinds* of vital lights as of living creatures, inasmuch as these lights are the very lives, souls, and forms of living things." So that, though the differences of their respective lives constitute all the differences among natural things, yet those differences may be both quantitative and qualitative.

With regard to the *origin of things natural*, we must believe that there are but three elements; for "in the beginning God created the heavens and the earth ;" and the earth was formed from water. The heavens, moreover, consist of air and water; therefore these two are the primary elements, and earth a secondary one. Water is the material substratum of all things; air, *æther*, or *aura vitalis*, indicates the vivifying principle.

Thus was matter formed, as water. But it had to be vivified by the union of an efficient cause with it. And the efficient or seminal causes were, and still are, scattered everywhere in profusion by the Creator, as *ferments.*† They are infinite in number and in kind;

* This meaning of *form* must be borne in mind, for the term will be often again used in the same sense; *shape* will imply the ordinary meaning of form.

† The term was not used by Van Helmont in its present meaning, as a substance to excite fermentation, but rather in the figurative sense in which we still sometimes use it; or, in general, as a *dynamic principle.*

there were and are as many of them as there are vital actions in the natural world; and they are all incessantly active, ever exercising their power upon the matter around them, and producing, as they did at first, minerals, and the seeds of even those plants and animals which are also capable of a more special propagation. Moreover, while the efficient cause of every living thing is transitory, ceasing to be with that which it enlivens, these special ferments are permanent, and can never cease to work, each in its peculiar way, upon common matter.

However, the new creature, whether its seed is formed by the action of a ferment on common matter, or by proper generation, is not perfected without the especial act of God.

"Animal does not generate animal, but the seed in order to the animal. And the seed is to the form of the animal as a disposing architect, but not as the maker of the form. The archæus is borrowed from the parent, but not the form, nor the light of life by which the form shines. (Van Helmont, quoted p. 10.)

"In every seed, indeed, there is a proper power, a special archæus, on which the type of its kind is impressed from the parent organism, (or is received from the special ferment;) and which has, and is capable of, all that is required for the formation of the new creature up to a certain point; but the essential form of the creature, which is one with its life, and in the higher beings is one with the perceptive mind (*anima sensitiva,*) the archæus cannot of its own power acquire. This is breathed into each individual being as the breath of life from God, as soon as by the action of the archæus in its seed it has attained a certain stage of development. And now first it possesses a form of life of its own; for hitherto it had been, in a measure, a part of another being, and had only a borrowed life. This is true not of plants and animals alone, which exhibit so distinct and determinate expressions of life, but also of the lowest creatures and even of the minerals; for though these do not propagate by seeds, yet they possess a determinate form of life, an *aura vitalis,* an archæus; for, of necessity, whatsoever is generated must have a disposer within it." (Spiess's Paraphrase, p. 11.)

Thus are all the individuals in nature generated. We come now to their *maintenance.* The inspiration of the determinate form of life, in the manner just described, is, in the case of the higher organisms, associated with an important change in the archæus of the new being:

"Hitherto the archæus was, to a certain extent, under the government of the parent life, whose product it was; but now it enters into the service of the new form of life, which, though it be in kind the same as that of the parent, is yet another. The archæus is now no longer a *causa efficiens,* acting of its own power and after its own type (though an impressed type;) but it becomes luminous, that is, it immediately illuminates, it becomes the servant of life, the sole and immediate witness, executor, and organ, as well as the lodging, of the life. Before, it appeared to us as power, as a dynamic principle, *in opposition to* matter; now it appears, in the higher organized and really animated beings, as *belonging to* the body or matter, and life enters in as a higher dynamic principle, determining the proper form over and above the archæus. The archæus is indeed still power, but it is the power inseparably connected with and constantly acting in the matter, and governed and directed by a higher and quite incorporeal essence, the life which is identical with the mind (*anima;*) in short, it is the corporeal servant and mediator of the life, and that by which all the exercises of life are executed." (Spiess's Paraphrase, pp. 14-5.)

Now, if these things be true, an archæus must dwell in every part; and every particle of the body of the more composite creatures must have its own archæus or power, working according to a model or to determined laws. And all these *archæi insiti* must be subject to the one *archæus influus,* which in its turn is subordinate to the life or the soul.

"Thus, in development, the archæus perambulates all the dark places and recesses of his seed, and begins to transform the matter after the fashion of his model. Here he places the heart, and there marks out the brain; and in each place of his own universal monarchy he appoints a permanent residing president, according to the need and the destinies of the parts. And that president remains curator and internal pilot (of the part) even to death; while the other, the chief archæus, lucid and never idle, moving about and assigned to no one member, keeps watch over the several pilots of the members." (Van Helmont, quoted p. 15.)

Throughout life the archæi are the proper agents of the vital processes. With regard to nutrition the old notion is erroneous, that out of the nutriment that which is appropriate is taken by each part, and that which is unfit is rejected; there is in truth a continual transmutation of the simplest matters by the power of the archæus in each part. As in the seed, the archæus forms all the organs from the one simple substance; so in the nutrition of each part, its archæus, according to its type, changes and fashions the common material, sap or blood, and assimilates it. Moreover, as every substance has its own archæus, it follows that it is not mere matter which is appropriated, but matter and an archæus; in other words, not inactive but active matter, or matter with peculiar properties and forces. And in this appropriation, either the archæus of the appropriating body, being the more powerful, subdues that of the appropriated; or, being the weaker, it is itself subdued. But in neither case does the weaker archæus lose all its proper tendencies; it still exercises an influence on the stronger, and in some measure alters its condition by the retention of its own properties. Hence are explicable the action of medicines, whose archæi are not annulled in the recipient, but retain some of their force and influence the stronger archæus—the influence of various soils on the same plant—the effects of the causes of many diseases, &c.

As to the *corruption* and *passing away* of beings, corruption is no mere negation or privation, as Aristotle thought; it is not a cessation, but a change. Neither does this corruption or change affect the form of any being, but only the matter, which passes into another form of life.

"Forms are not ever corrupted; though, indeed, they cease to be (*intereunt.*) The human spirit alone passes away, safe and whole; all other forms perish. But matter never passes away, nor ceases to be; it is corrupted. And so corruption is of matter alone.......Corruption therefore is a certain disposition of matter deserted by its weary pilot, Vulcan (its archæus.) For so long as a body sails with its pilot in good plight, it listens not to alien ferments." (Van Helmont, quoted p. 21.)

Not that the archæus gives way of itself, but only that it falls under the attack of some other ferment; and then corruption, beginning with a substitution of one ferment for another, goes on till it has attained its end.

B. SPECIAL PHYSIOLOGY. These general considerations imply and guide to many doctrines in special physiology different from those which the ancients have held.

*Natural functions.* In regard to these the ancients held that *diges-*

*tion,* in which is included everything from gastric digestion to nutrition and excretion, consists in three stages, viz. a first, or *concoctio ventriculi,* taking place in the whole digestive canal, and having for its product the chyle, for its excrement the fæces; a second, or *concoctio in jecore,* taking place in the liver, its product being the blood, its excrement the urine (which they did not distinguish from the serum,) and the yellow and black bile; and a third digestion, or *concoctio in partibus singulis,* of which the organ is the whole body, its product the four secondary humours, four degrees of assimilation of the blood, and its excrement the sweat.

Now, in the first place, heat is not, as the ancients maintained, the prime cause of the first digestion; it is an excitant, but not an efficient, of digestion. The true author of digestion is "a certain vital faculty, a ferment it may be called, which truly and *formally* transmutes the food." Instead of three there must be reckoned six stages of digestion, each of which has its proper organ, in which the change is effected by a proper ferment or indwelling vital force acting in it, not in a material, but in a formal dynamic manner. Each ferment is indeed united to a certain matter, as the *fermentum stomachi* to the *acor* of the stomach; but it is the ferment, not the matter, which is the real agent in the process.

*The first digestion* is the gastric, in which the gastric ferment converts all the food into chyme.

*The second digestion* is in the duodenum, in which the ferment of the bile changes the chyme from *sal acidum* to *sal salsum.* The bile is not, as hitherto supposed, mere excrement; but "a noble and vital viscus," the bearer of a vital ferment. In this stage also the watery part of the food is separated, and, with various salts, passes to the kidney as urine; and the indigestible parts become fæces.

*The third digestion* begins in the mesenteric veins, and is especially effected in the veins of the liver, whose ferment changes the chyle into *cruor.* This is the only office of the liver, and the old notions of the formation of yellow and black bile and urine in it are absurd. Neither is the liver nourished, as has been supposed, from the cruor, nor the stomach from the chyme, nor each organ from any substance carried to it, as Fernelius imagined; but the food must have passed through every stage of digestion before it is fit for the nutrition of any part.

*The fourth digestion* is in the heart, whose ferment makes the thick dark blood of the vena cava brighter and more volatile; changing *cruor* to *sanguis.*

*The fifth digestion* transmutes the arterial blood into the vital spirit of the archæus; and in neither of these two is any excrement produced.

*The sixth digestion* is nutrition proper. "It is perfected in the several kitchens of the members; and there are (for it) as many stomachs as there are fed members. In it the spirit indwelling in each part digests for itself its own aliment." There are not four stages only to assimilation; there may be hundreds before the blood is changed into a solid part.

It is to be noticed that the serum (*latex* or *latex humor*) is not, as is commonly supposed, either mere urine or sweat, or any other mere excrement, but a proper product of the formation of blood; and it serves to dilute and moderate the cruor, to dissolve the salts received for excretion, as well as the nutritive material, and to contribute to the formation of all the watery excretions, as well as of the sweat in which it ultimately passes off; each organ, by its proper ferment, forms from the serum its peculiar excretion. Moreover, by its unhealthy changes, or by injurious materials added to it, the serum is a frequent excitant of disease.

*Of the vital functions,* (circulation and respiration.) There is no *spiritus naturalis* or *spiritus vitalis,* such as the ancients have imagined to be separate existences formed in and moving with the blood; nor any *spiritus animalis,* such as they imagined to be sublimed in the brain. The cruor generated in the liver possesses itself the properties hitherto ascribed to the *spiritus naturalis* supposed to be formed with it. And when the *cruor* is, by the ferment of the heart, converted into *sanguis,* and is in a measure enlightened by the soul, it becomes itself the *spiritus vitalis,* the carrier of life to all the parts of the body; not however, by its material composition, but by its being enlivened and inspirited by the soul. And as to the *spiritus animalis,* its supposed offices, sensation, motion, and the higher actions of the mind are due to the one *spiritus vitalis,* which is capable of assuming the character of each of the parts to which it is sent, and of presiding over their several functions. That which goes to the tongue exercises taste; but the same spirit in the fingers does not taste, but feels.

Again there is no *calor innatus* in the heart, such as the ancients supposed to change the natural spirit or vapour of the blood into the vital spirit; nor, consequently, do the pulse and respiration serve for the cooling of this heated spirit. The fuliginous excrement of the spirit also, supposed to be sent from the right heart to the lungs and discharged in expiration, as well as from the skin at each contraction of the arteries, is a mere fable; and so is the nutriment of the spirit supposed to be derived from the atmospheric air drawn into the blood in inspiration and in each dilatation of the arteries in their pulses. The true purposes served by the pulse and the heart's action and the motion of the blood are, "1, the translation of the cruor from the sinus of the vena cava to the left cavity of the heart; 2, the increase of heat; 3, the fabrication of arterial blood; 4, the production of vital spirit; and 5, the distribution of the primordial life residing in the spirit of the heart through the whole body." And in respiration are effected, 1, the evaporation of watery vapour (not smoke) formed in the conversion of cruor into sanguis in the heart; 2, the entrance of air and all it contains, including often the causes of disease, into the body.

*Animal functions.* Besides sensation and motion, which almost alone the ancients include, in this class, there are other functions which they have nearly neglected, which are observed only in the higher organisms, and by which the connexion and oneness of the phenomena of life are maintained. Every natural being is the expression of *one* indwelling idea. The foundation of this unity is the life itself, which in animals is identical with the sensitive mind, the *anima sensitiva,* governing every single function and inducing their agreement and unity. But this life or light cannot act directly upon matter, except when the latter has a dynamic principle in it; and on this, the archæus, the life acts. In the composite animals, where each part has its archæus, the *archæus influus* is over all, and is at the same time the medium between mind and body. Through the medium of the superior archæus the mind governs all parts of the body, and by its vital ray is everywhere present and president.

But this dependence of all the parts on one central point does not completely constitute their unity. They are further combined; first, by their mutual dependence one upon another, each preparing or doing something necessary for the rest; and, secondly, by the influence which the alteration of one part has upon the functions of another, as shown by observation of facts: (in a word, by sympathy.) The ancients could not explain this, for they supposed that something material must connect the sympathising parts, or pass from one to the other. But the sympathy depends on a peculiar mode of action of the whole economy, an *actio regiminis* or *influentialis*, which works without any corporeal contact whatever—by a *continuation of virtue*—as the stars act upon each other and upon terrestrial objects, and as magnetism and sympathy (in things out of our bodies) work. Hence are dispelled all the *vapours* which the ancients supposed to pass from one part to another.* They are all results of the *actio regiminis*, in which, as the soul by a nod indicates its will to any part, so the state of any part is conveyed dynamically and through the medium of the archæus to another part in which corresponding changes of action are induced.

c. Psychology. The true key to the whole of Van Helmont's system is furnished by psychology, and its principles are these:†

The spirit of man is the only part really created in or after the image of God, and it is therefore immortal and unchangeable. The mind is but as the shell of the spirit (*siliqua mentis;*) it belongs to the earthy transitory life, and all its functions,—perception, memory, induction, judgment, discourse, imagination, fancy, are under the strict laws of necessity to which all nature is subject.

"At first there was an immediate connubial union of the immortal spirit with the archæus......Before the fall of Adam there was no sensitive mind in man. ......But after the fall the soul withdrew itself, like a nucleus, into the centre of the sensitive mind, to which it was then bound by the chain of life." (Van Helmont, quoted p. 59.)

The understanding and discursive faculty shine not obscurely in the brutes; and these faculties are therefore not to be held at so high an estimate as they have been hitherto. The mind, indeed, is not itself corporeal, nor the product of the body, nor matter; but all its functions are the results of life working in that which is corporeal, in the same manner as all the phenomena of life are the acts of matter and a power, the archæus, which again is, strictly speaking, only a part of life. Nay, "life and the mind are [in brutes], as it were, synonymous." But

* Van Helmont is unusually rich in his facts regarding this sympathy, especially that of other parts with the stomach and uterus; he even speaks of influences *reflected* from the uterus.

† In the following section, that which Van Helmont calls *mens* and Dr. Spiess *geist*, we translate *spirit; anima*, *anima sensitiva*, and *Seele*, we render *mind*, or *sensitive mind; ratio* and *discursus* and *intellectus*, (which, with Van Helmont, are synonymous,) and which Spiess renders *verstand*, are, *understanding;* in Coleridge's meaning, "the faculty judging according to sense." We would particularly draw attention also to the frequent similarity of Coleridge's and Helmont's views.

neither of them is self-existent; the spirit alone is truly subjective. The mind can of itself originate nothing; all its knowledge is acquired through the senses; its thoughts are all sensuous, and "the more thinking approaches towards abstract reasoning, the more does it partake rather of the life of the soul than of the peculiar vitality of the sensitive faculty." (Van Helmont, quoted p. 63.)

There is, however, a great difference between the mind or life of the brute and that of man. Both are imparted by divine in-breathing in the formation of the creature, as already expressed. But the mind is the *whole* and *sole* life of the brute, all whose functions depend on it; and when it has accomplished all it was ordained to do, it "passes away into nothingness like the light of a taper." It is otherwise with the mind of man; he has an undying spirit, which, after the death of the body, exists for ever as a *substantia luminosa.* As in brutes the mind, so in man the spirit, is the true light of life. His mind (which he first acquired in the fall) is indeed also a vital light—all the vital acts of the body depending on it—but it is not the life itself; it borrows its life from the spirit, as the moon from the sun.

"And as in the moon the light of the sun loses its warmth, and puts on a coldness foreign to itself; so also in the sensuous life (or mind) the ray of the spirit, though in its purity it is intellectual, passes into the domain of the sensuous, and finds there a terrene law opposed to the law of the spirit." (Van Helmont, quoted p. 66.)

There are thus three stages: 1. In the lowest stages of life the archæus is the life or light, and acts of itself, though according to laws impressed upon it. 2. In the higher, that is in brutes, the *anima sensitiva* is the life; it is the determining and form-giving power, and the archæus is its servant. 3. In the highest, in man, there is an immortal spirit, clothed upon and obscured by the mind, through which it imperfectly acts, and which again acts through the archæus.

Now, since the mind's influence is felt in every part of the body, and the mind or life is not divisible (though parts of the body may be removed without damage to it,) it follows that the mind must have some proper central domicile. "As the sun is not truly anywhere but in its own place in the heavens, though its light is wherever it looks; so is the judgment of the mind given from a central place." This place cannot be the heart, that is too restless; nor the brain, for many reasons. It is the *duumviratus*, the stomach and the spleen, which belong essentially to each other; for "the spleen serves to prepare the ferment of the stomach, and is its sun, its concoctor, and director; and therefore the conspiring of the two viscera shall be called *duumvirate*." These are to the animal what the roots are to the plant; and as in the latter the beginning or principle of life is in the root, so in the animal must it be in the region of the stomach. Besides, thoughts are said to rise *to* the heart, not to arise *in* it, as if they were engendered in it; nor to descend to it, as if they came from the brain. And, again, the workings of the passions are felt most in the precordia. But the best evidence I (Van Helmont) gained when in an extasy under the influence of napellus: "I *felt* that nothing is done in the head; and that, in an unspeakable manner, the whole mind thinks in the precordia."

From this its residence which it never leaves, the mind radiating, is, after a manner, present everywhere. But, since it is not a pure spirit, but only the spirit operating through the sensuous faculty, it cannot of itself generate or do anything, but requires for every act a distinct organ. This is true of the properly mental acts as well as of the corporeal already considered. Each of these has its proper organ. The will has its seat in the heart; the organ for the mental acts, and especially for all those of objective thought, is the brain ; not, however, that the brain can of itself think anything, but only because in it the memory has its seat. The true agents of thought are the mind and the spirit within it in the precordia, whence their light radiates. The brain then is, through the afflux of rays from the precordia, the organ of all thoughts of objects (all subjective thought is spiritual and precordial;) it is also a kind of record-chamber for the thoughts of the mind: "in regard of motion it is the executive organ of the mind, presiding over the nerves and muscles; in regard of sense it possesses in itself the faculties of memory, will, and imagination." (Van Helmont, quoted p. 73.)

As for the spirit, "it is a spiritual *substance* [purely subjective;] a vital luminous creation."

"Its continuous and undisturbed operations are insensible; for that which in itself is sensible cannot be altogether spiritual and merely abstract......Happy the man to whom it is permitted to perceive those insensible operations of the spirit, and to reflect on them against and above the powers of the sensuous mind ! ......Would that it could be granted to an atheist, at least for a single moment, to taste what it is to reason rationally, that he might, as it were by touching, feel the immortality of the spirit!......The proper operations of the spirit cannot be more distinctly propounded than by the speech of silence; since that which is most properly the genial operation of the spirit is altogether abstract and is believed......The spirit is the immediate image of divinity ; therefore, as the eye beholds nothing more absolutely than the sun himself, although it cannot tolerate his brightness, and beholds all other things by him ; so the spirit, properly, principally, and intimately, thinks on and contemplates nothing but that divine unity, and all other things by it......But who am I who write these things? Verily I fear lest I should be the bell calling the faithful to the temple, but itself remaining out of doors in the height of the tower." (Van Helmont, quoted pp. 75-9.)

d. General pathology. "Disease and death are diametrically opposed to life. Hence, every disease must implicate the life itself, and since it can do this only by coming into contact with it, it follows that every disease must have its seat in the life itself. But since the real vital principle, which is identical with the mind, is incorporeal, and acts on the body only through its organ the archæus, therefore it is the archæus through which every disease manifests itself, and which is the proper seat of all disease. That which makes the unity of all the parts is the life acting through the archæus; this is altered in disease; and in it therefore must be the seat of all disease. The mind has its seat with the archæus, in the precordia, and here also must be the head-quarters of disease.

"But what is *disease?* It is not a mere negation, a mere absence of completeness or of health, but something positive; it is not a diathesis, nor an accident disturbing actions, much less is it that disturbance itself resulting from the conflux of injurious causes with our corrective powers. But disease is an *ens reale*, having a material and an efficient cause, excited by occasional causes." (Van Helmont, quoted p. 84.) It is "an entity verily subsisting in the body," and working according to definite laws; and this may be proved especially by the phenomena of periodical and latent diseases, in which the disease continues to exist even when there is no disturbance of function.

Disease then is a peculiar form (or mode) of life; and cannot, therefore, exist in the same point with the proper life, i.e., with health. And since every life works according to one determined *idea,* (or according to definite laws,) which is impressed upon the archæus,(or, in animals, upon the archæus influus, and through it upon the archæi insiti;) it follows that when disease (whose consequence is a disturbance of the vital acts, and whose seat is in the life or in its organ,) ensues, it must be because the archæus has had some new foreign *idea* (or model of action) impressed upon it. And this new idea may be impressed on either the life or mind; but more generally it is impressed on an archæus influus, or an archæus insitus.

It has been said that in every disease there is both a material and an efficient cause; and again, the archæus (in the animal body) is no mere dynamic essence, but a power so inseparably connected with matter, that an alteration in the one is of necessity immediately followed by a change in the other. Hence, either the material or the efficient cause of disease may act upon the archæus; that is, either the matter with which the archæus is united may be primarily changed, or the archæus itself may have a morbid idea impressed upon it; in other words, the cause of disease may be either dynamic or material. It is to be understood also that though diseases are in so many respects analogous to other forms of life, yet they differ in this; that they can manifest themselves only in other living organisms; they have no self-standing life. They are archæi acting after a wrong plan; and their symptoms are the phenomena consequent on this wrong acting.

Disease originates whenever any noxious impression is made upon an archæus; and this, whether the impression be entirely dynamic, or the result of a noxious matter either from without, or generated within the body. By this the archæus is excited, frightened, or disturbed; and under this disturbance the morbid idea is generated. This is always the first step; and next, but immediately, the idea (like every idea of the archæus,) incorporates itself, as the *causa efficiens seu seminalis morbi,* in some adjacent or appropriate matter, and thus attains a definite existence as actual disease. Diseases are thus produced like other beings, by the incorporation of an idea in matter; but with this difference, the ideas of other natural beings are embodied in seeds at the creation, or exist as ferments at all times; while the *ideæ morbosæ* are first generated in the archæus of a living being and can only in some cases be propagated.

The occasional causes of disease are all those things which are prior to the incorporation of the morbid archæal idea. Wounds, poisons, noxious food, contagions, &c. are of this kind. However intimately they may have passed into the body, they are yet *external* causes; they do not constitute the disease; nor do they produce it till they have excited the archæus, and engendered the morbid mode of action. So also the products of a disease; they are not the disease, though by their noxious influence they may be the occasional causes of secondary diseases. These products moreover, or material changes of structure or composition, are produced when an archæus insitus is affected, and this may take place either directly or through the archæus influus; so long as the latter alone suffers, symptoms of disease and mere disturbances of function are alone produced.

Thus originating, diseases in general are of two kinds. 1, Those which have their morbid idea in the archæus influus, which are general and transitory, or, for the most part, acute diseases; and 2, Those in which the idea is in an archæus insitus, which are local and persistent or chronic.

Another mode of division of diseases is according to their origin, in which view all are either, 1, Archæal, (properly so-called,) in which the morbid idea *originates*, (in some unknown mode,) in the archæus; or 2, Archæal, but secondarily so; the morbid idea being generated by something troubling the archæus.

Of the diseases which are archæal in their origin there are four kinds. 1. Hereditary diseases, in which, in generation, a morbid idea is impressed upon the archæus of the seed, and remains for a certain time, sealed up in it. 2. Silent or latent diseases, such as epilepsy, which have no material foundation, and in which the morbid idea lies long at rest and concealed in the inmost part of the archæus. 3. *Torturæ noctis*, periodical diseases, which manifest themselves chiefly at night, and seemingly, under the influence of the moon. 4. Those due to *robur inequale*, in which, by an hereditary or an acquired defect, the vital force is imparted more or less abundantly to one than to other parts of the body; as one often sees, in whole families, a particular organ weaker, and, though not diseased, yet more prone to disease than the rest.

Of the diseases which originate in occasional causes disturbing the archæus there are many more kinds, but they may be divided, in general, into two great classes; those namely of *recepta* and *retenta.*

The *recepta* are always primary diseases; they result from noxious influences which get into the body as it were by stealth, and disturb the archæus till a morbid idea is generated. And these influences are of four kinds, viz. 1st, *injecta a sagis*, such as are thrown in by witches and charms: 2d, *concepta*, those which have their origin in disturbances of the passions and affections, which, thus disturbed, communicate a morbid idea in the archæus, and through it produce diseases of the body as well as of the mind: 3d, *inspirata*, which are taken in with the air, such as all vapours, and which act, not upon the lungs, but, by penetrating the diaphragm, upon the stomach: and, 4th, *suscepta*, such as act mechanically, as wounds, blows, &c.; but these are causes of disease only when the archæus is excited for the reparation of the injury.

The *retenta* are always secondary diseases; the noxious influence which disturbs the archæus being the product of a previous disease, or something which should have been removed from the body, but has been retained through a diseased state of the part destined to remove it. The noxious influences may be placed in two divisions. 1. *Assumta*, in which matters imperfectly digested or assimilated in consequence of disease of the digestive organs, act injuriously on the archæus, whether they be food, drink, poison, or physic. And in this class also are the products of former diseases. 2. *Retenta innata*, which again are divisible into three kinds, viz.—*a*, *Retenta relicta*, when, through previous disease, the various secretions and excretions of the several stages of digestion, instead of being in due time cast out, are left in the body; *β*, *Retenta transmutata*, in which morbid matters, or defective compositions of the fluids, are produced by previous disease and pass into the blood; *γ*, *Retenta transmissa*, when the *transmutata*, instead of being excreted, pass to parts of the body to which they are not properly destined.

E. General therapeutics. The philosophy of healing is consentaneous with that of all this system. God, in his goodness, has given remedies for all diseases; and it is our place to know and use them. The old notion of *signatures*, or likenesses, as indications of remedies is mere nonsense: man is created after the image of God, not of nature.

Medicines, like all other things, may act in two ways, viz. corporeally, as all purely chemical and mechanical means; and abstractedly or formally, by a pure dynamic quality. The general properties of medicines may be named *savours*, not because we can taste them, but because all organs seem to possess a sense by which they can discover whether anything that comes in contact with them is friendly or hostile. In this sense there are two kinds of savours: 1, the *sapores rerum*, by which things are bitter, sharp, salt, &c., or, in general, *salia;* and, 2, the specific, formal savours, by which their intimate essence is revealed, though not to our senses. In medicine, the material savours cannot act on the disease itself, which has its seat in the immaterial life; they can only correct some of the causes of disease, such as the noxious substances generated in the modes already mentioned. In this manner some act at once in the stomach; others, such as diuretics, must first pass through the second digestion; both, by removing the occasional cause of the disease, give opportunity for the archæus to act.

But the most important are the medicines which act by a specific formal power; and to know these is the physician's highest attainment. That there are such medicines which act, not by any material combination, but by their mere presence, their contact, or, as it were, by a glance, is proved by many facts: e. g. water in which mercury has been boiled kills worms, though the mercury has lost no weight in boiling. Their real mode of operation cannot, indeed, be discerned; for even the specific savour or odour by which the archæus knows that they are beneficial, is but an evidence of their virtue, not the virtue itself; and even this savour is not cognizable by our senses. The active power is an entirely hidden property, identical with the essence of that to which it belongs.

The whole of our knowledge of the action of medicines may be thus expressed:

"Every remedy acts immediately and principally upon the archæus of the stomach; and the archæus in succession acts according to the disposition which he imbibes, and which is generated in him by the virtue of the remedy. Further, also, it follows that every remedy whatever acts by approximation, after the manner of a mirror; since above and beyond its material composition it acts by mere approach to the archæus. Now, the archæus himself first perceives the virtue imbibed from the remedy, and in that sensation forms for himself an idea of things to be done by him in following the dispositions of that virtue; whence, consequently, engendering in himself peace or rest or anger, and, assuming the ideas of these, he presently disperses them among the viscera which are listening to the

*actio regiminis,* performing agreeable or opposed services according to the command of the vital archæus." (Van Helmont, quoted p. 144.)

III. We have thus, in the briefest possible space, given a general view of the famous system of Van Helmont; and little room is left for the other department of the work. However, the chief points in which this system is distinguished from those of the Greek and Arabian schools have been noticed in the course of the analysis; and for those in which Van Helmont differs from Paracelsus, a comparison of this article with that on the works of the latter will afford a sufficient clue. It cannot be denied that Paracelsus had imagined the foundation of Van Helmont's system; yet he had expressed himself so obscurely, and had surrounded his truth with so much error, that re-invention would have been nearly as easy as a comprehension of his works. Besides, his followers had so misunderstood and perverted his views; they had so neglected that which was good, and made so ill an use of that which was already bad; and his school had degenerated into such a mere cabal of mad fanatics; that when Van Helmont came into the field the doctrines of the schools were almost as universally received among the more learned of the physicians as if Paracelsus had never lived. The whole battle had to be fought again; and Van Helmont's victory was lasting. To him belongs all the honour of setting in motion that scientific inquiry into the laws of life as of a peculiar principle, which has now led to the discovery of many laws and a doubt of the existence of the principle. We call it scientific, though in Van Helmont's time the inquiry was conducted in a manner which would not now deserve that epithet; but, considering the state of knowledge when he wrote, the absence of all certain principles in all those which are now sciences, the volumes of falsehoods universally admitted as truths, the cramped and fettered modes of reasoning which were then in vogue, and the difficulties of inquiry of every kind, we cannot but wonder at the success which Van Helmont attained. His system, in the midst of all its error, contains the elements of numerous great truths; divesting it, even more than we have done, of its strange words, it might be read as a work written but half a century ago. For archæus, read nature or vital principle, and it would in many parts describe very fairly the popular medical theory of the present day. And, on the other hand, there is no weak resemblance between its metaphysical part and that which is now generally current in the German idealism-schools: with a few mutual adaptations of mere expressions the two systems might be made essentially alike.

To all who are interested in the history of medicine, Dr. Spiess has rendered a truly admirable service. With enormous labour, he has given a clear and intelligible view of a system which, in the original, is most obscure: he has distinctly and briefly interpreted that which has hitherto been utterly misrepresented; and he has rendered due honour on just grounds to one who, if he has hitherto at all received it, has received it from those who were but imperfectly acquainted with his merits. That, in such a task, the learned interpreter should have given too bright a view of his author, should have often put aside or veiled his faults, and have passed by his deep obscurities, is but a venial fault.

